# Microstructure and Porosity Evolution of the Ti–35Zr Biomedical Alloy Produced by Elemental Powder Metallurgy

**DOI:** 10.3390/ma13204539

**Published:** 2020-10-13

**Authors:** Izabela Matuła, Grzegorz Dercz, Maciej Zubko, Joanna Maszybrocka, Justyna Jurek-Suliga, Sylwia Golba, Izabela Jendrzejewska

**Affiliations:** 1 Institute of Materials Engineering, University of Silesia in Katowice, 75 Pułku Piechoty Street 1 A, 41-500 Chorzów, Poland; maciej.zubko@us.edu.pl (M.Z.); joanna.maszybrocka@us.edu.pl (J.M.); justyna.jurek-suliga@us.edu.pl (J.J.-S.); sylwia.golba@us.edu.pl (S.G.); 2 Department of Physics, University of Hradec Kralove, Rokitanskeho 62, 500-03 Hradec Kralove, Czech Republic; 3 Institute of Chemistry, University of Silesia, Szkolna 9, 40-006 Katowice, Poland; Izabela.jendrzejewska@us.edu.pl

**Keywords:** Ti–Zr alloys, biomaterial, powder metallurgy, porosity

## Abstract

In the present study, the structure and porosity of binary Ti–35Zr (wt.%) alloy were investigated, allowing to consider powder metallurgy as a production method for new metallic materials for potential medical applications. The porous Ti–Zr alloys were obtained by milling, cold isostatic pressing and sintering. The pressure during cold isostatic pressing was a changing parameter and was respectively 250, 500, 750 and 1000 MPa. The X-ray diffraction study revealed only the α phase, which corresponds to the Ti–Zr phase diagram. The microstructure of the Ti–35Zr was observed by optical microscopy and scanning electron microscopy. These observations revealed that the volume fraction of the pores decreased from over 20% to about 7% with increasing pressure during the cold isostatic pressing. The microhardness measurements showed changes from 137 HV0.5 to 225 HV0.5.

## 1. Introduction

The demand for novel metallic biomaterials is increasing year after year in terms of an ageing population with the expectation of a high quality of life [1,2]. Nowadays, conventional metallic biomaterials such as commercially pure titanium (CP Ti) and Ti–6Al–4V ELI (Ti64 ELI) alloy are widely use in medicine as implants. The CP Ti and Ti64 ELI alloys exhibit mechanical properties, which are different in terms of properties of bone. Additionally, in the case of Ti64 ELI, the toxic vanadium (V) ions released from metal implants severely affect the long-term biocompatibility of these alloys [2,3]. Also, aluminum (Al) ions are neurotoxic and inhibit bone mineralization [2,4,5,6,7,8]. Thereby, scientists focus on the study of Al- and V-free titanium alloys [9,10,11,12,13,14].

It is well known that the properties of Ti alloys are sensitive to their phases structure. This is important because the phases may be stabilized by the addition of appropriate alloying elements. Thus, the mechanical properties of titanium might be enhanced by alloying [15,16]. A good candidate for alloying is Zr. Titanium and zirconium have been extensively investigated as biomedical materials due to their similarity in properties because both Ti and Zr have the same crystal structure and belong to the same group in the periodic table of elements. The system Ti–Zr has a hexagonal structure closely packed with the (0002) plane. The phase diagram revealed the complete solution over the whole range of components [17,18]. Both elements have similar chemical and physical properties, such as low density, good mechanical properties, high melting point, high corrosion resistance and excellent biocompatibility, which is attributed to a stable surface oxide layer [19]. Also, it has been confirmed that both elements are non-toxic and promote osseointegration [20]. Thereby, it is a material of interest for surgical implants with superior corrosion resistance compared to most other alloy systems [21,22,23,24]. In the literature, there is evidence for the good osseointegration of zirconium implants in vivo [21,25,26], and studies comparing zirconium and titanium implants showed that the degree of bone–implant contact is higher in the case of zirconium [21,27]. Another advantage of alloying zirconium to titanium is that the phase transition of the alloy is lowered with an increase in the amount (max 55 wt.%) of zirconium [28]. Reducing the phase transition temperature of Ti–Zr alloy increases the capabilities to change properties and microstructure.

The major problem of using metals for implants that their Young’s modulus is too large in comparison to the properties of human bones. The Young’s modulus of α (pure Ti) and α + β (Ti–6Al–4V) titanium alloys, respectively, 105 GPa and 110 GPa, are about three times higher than that of bone (30 GPa) [11,12]. This mismatch induces stress between the implant material and natural bone, which can cause damage to the tissues and premature failure of the implants [13]. It should be emphasized that the Zr–Ti alloys exhibit elastic modulus ranging from 68 GPa for Zr–30Ti alloy to 78 GPa for Zr–40Ti. The Zr–Ti alloy with 30 wt.% Ti content shows the lowest bending modulus, and it had a significantly lower (*p* < 0.05) bending modulus than Zr–20Ti and Zr–40Ti alloys [29]. The hardness of c.p. Ti was 186 HV. Generally, the hardness value of the Ti–Zr alloys increased with increasing Zr content, for example for Ti–10Zr ranged from 266 HV and 350 HV for Ti–40Zr [20]. In Ti–Zr alloy, adding up to 50 at% of Zr an increase in hardness of approximately 2.5 times of pure titanium can be obtained. This phenomenon is an effect of solid solution strengthening caused by the difference between the atomic radii of Ti and Zr (1.47 and 1.62 Å, respectively) [20,29,30]. Another way to further reduce Young’s modulus is to develop new porous titanium alloys containing non-toxic and non-allergenic elements, thereby minimizing damages to bone tissues adjacent to the implant and, prolonging the device lifetime. The powder metallurgy (PM) method combined with sintering in a strong vacuum has been widely used, producing porous structures and also protecting against oxidation of the material. Pores are formed from the interstices of powder particle arrangements [31]. The PM production method allows to control pore size, shape, orientation and distribution, including the creation of hierarchical and functionally graded pore structures. PM has been successfully used in the production of several titanium alloys and appears to be an ideal method for the preparation of porous materials [20,32,33,34]. Moreover, the porous material promotes bone ingrowth into the pores and allows stress to be transferred from the implant to the bone [10]. In view of the stability of the implant, a material should be of high porosity and the preferred pore size for osseointegration is from 50 micrometers to a few hundred micrometers. In addition, pores should be connected together, which allows the new bone tissue to penetrate into the material and enables the flow of body fluids [14,15,16]. The presence of pores can lower the Young’s modulus of the material [10,15]. This is the reason why in the production of biomaterials scientists focus their attention on the porosity of the material.

Based on these data and previous experience about the properties of alloys of Ti–Zr, the authors have proposed a new porous Ti–35Zr (wt.%) alloy composition. The research is based on literature reports on materials used for implants and, in particular, on the porosity structure suitable for improving metal–bone osteointegration. The produced material is supposed to be a hypothetical starting point for the material for long-term bone implants. The aim of the undertaken studies was the production of porous Ti–35Zr alloy by the powder metallurgy method, combined with annealing in vacuum, and to investigate the effect of the isostatic cold pressing on the structure, porosity microstructure and microhardness. The result of the carried analyses will be a starting point for further material development and extended research.

## 2. Materials and Methods

Commercial powders of Ti (Atlantic Equipment Engineers (AEE, Upper Saddle River, NJ, USA), purity 99.7%, particle size < 20 μm), and Zr (Atlantic Equipment Engineers (AEE), purity 99.5%, particle size < 300 μm), were used for the synthesis of the alloy as the initial materials. The elemental metal powders with a nominal composition of 65 wt.% titanium and 35 wt.% zirconium were blended in a high-purity argon atmosphere (≥ 99.99%) thoroughly at a rotation rate of 100 rpm for 3 h in the planetary ball mill Fritch Pulverisette 7 premium line. The material was prepared without any substances (e.g., space holders) which improve the porosity. The changing parameter was the pressure during cold isostatic pressing after blending and were respectively 250, 500, 750 and 1000 MPa. The samples were labelled TZ-250, TZ-500, TZ-750 and TZ-1000 according to the pressure used. Then, the green compacts were sintered in a strong vacuum (10^−4^ Pa) at 1000 °C for 24 h and cooled with the furnace to room temperature.

The phase contents of the obtained materials were studied by the X-ray diffraction method. The refinement of the X-ray diffraction pattern to assess the crystal structure was carried out using the Rietveld’s whole X-ray profile fitting technique with the DBWS 9807a program [35]. The pseudo-Voigt’s function was used to refine the observed ones to the calculated diffractograms [36,37]. The weight fraction of each component was determined based on the optimized scale factors with the use of the relation proposed by Hill and Howard [38].

The morphology of the initial powders and microstructure of sintered materials were observed by the scanning electron microscope (SEM) JSM 6480 (JEOL, Tokyo, Japan) with the accelerating voltage of 20 kV. Chemical analysis was performed using the energy dispersive X-ray spectroscopy (EDS), (IXRF, Austin, TX, USA) using the standard calibration method. The electron backscattered diffraction (EBSD) measurements were performed on the JSM 6480 SEM microscope equipped with Nordlys II EBSD detector from HKL company (Hobro, Denmark).

The specimens for observation were ground and polished by standard metallographic procedure. The analysis of three regions of a total actual area of about 1 mm^2^ for each sample was performed using ImageJ (1.8.0_172) image processing and analysis software. The planimetric method was used to calculate porosity and size of the pores using the following stereological parameters: the surface area of the pores $a_{p}$ (µm^2^); volume fraction of the pores V_V_ (%); and Feret’s diameter (largest distance between two points along the selection area). The shape of the pores was also assessed by the circularity

(1) $$f_{p1}=\frac{4\;\times \;\pi \;\times \;F}{L^{2}},$$

where *F* and *L* denote the area and perimeter of the analyzed object). Further, the porosity was assessed using the Archimedes method (as per the ASTM C373-88 standard). As a complementary test, measurements of surface area by the Brunauer–Emmett–Teller (BET) method with the use of nitrogen gas were carried out. The measurements were performed by Gemini VII powered by Micrometrics Instrument Corp (Norcross, GA, USA).

The analysis of the grains and subgrains size and shape was carried out on the images of the sample actual surface of approx. 0.1 mm^2^. To assess the microstructure, the following parameters were used: grain section surface area $a_{g}$ (µm^2^); grain size change ability factor

(2) $$v=\frac{\sigma_{x}}{a},$$

where *σ_x_*—grain size standard deviation; *a*—grain mean value; number of analyzed elements per the area unit of the image—N_A_ $\left(\frac{1}{{\mathrm{mm}}^{2}}\right)$.

The microhardness measurement was carried out as the first look at the mechanical properties of the obtained material. Vickers microhardness measurement was conducted with the load of 500 N for a loading time of 10 s on the microhardness tester 401MVD (Wolpert, Worcester, MA, USA).

## 3. Results and Discussion

**Figure 1:**
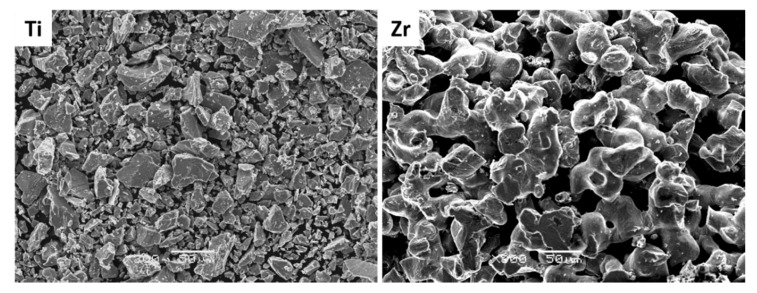
Scanning electron microscope (SEM) micrographs of Ti and Zr initial powders—scale bar 50 μm.

In Figure 1, the SEM micrographs of initial metal powders are present. The titanium powder morphology is irregular with sharp corners. The zirconium powder has larger particles, in addition they are joined into agglomerates. The nominal composition Ti–35Zr (wt.%) was designated so as to balance the strength, hardness and Young modulus for binary Ti–Zr alloy based on the literature. The materials for long-lasting implants should combine the high strength and the lowest possible Young modulus. The hardness and strength of Ti–Zr alloys increase with increasing content of Zr up to 50 wt.%, with relatively acceptable calculated values of Young Modulus [19,29,39,40,41].

**Figure 2:**
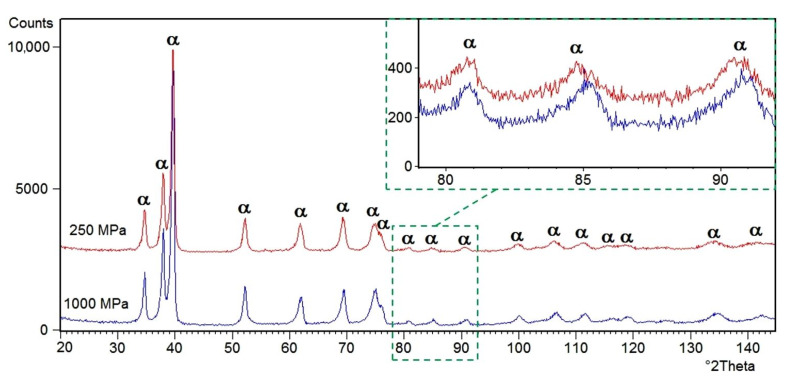
X-ray diffraction patterns of the material prepared with using different pressure during production.

The X-ray diffraction patterns of Ti–35Zr (wt.%) alloys are shown in Figure 2. For the Ti–35Zr alloy, all the diffraction peaks matched well with those of the α phase (ICDD PDF 00-044-1294). This study reveals that the material possesses structure of the α phase, which corresponds to the phase expected from the phase diagram. The Ti–Zr system shows a completely solid solution for both the high-temperature beta phase and the low-temperature alpha phase [28]. The β phase or any intermediate phases were not observed in the recorded XRD patterns. It should be mentioned that the Zr is a neutral alloying element added to a solid solution with titanium because it has an identical allotropic transformation with similar temperature of phase transition [28,29,42]. Previous research [43] has shown that the addition of Zr causes an increase of lattice parameters of the α phase in titanium alloys, due to the larger atomic radius of Zr (1.616 Å) than Ti (1.475 Å) [40]. The results of the refinement using Rietveld’s method confirmed that the addition of Zr causes distortion in an increase of the lattice parameters of the unit cell (Table 1). The change of pressure during manufacturing of the material decreases the unit cell parameters for all of the samples.

**Figure 3:**
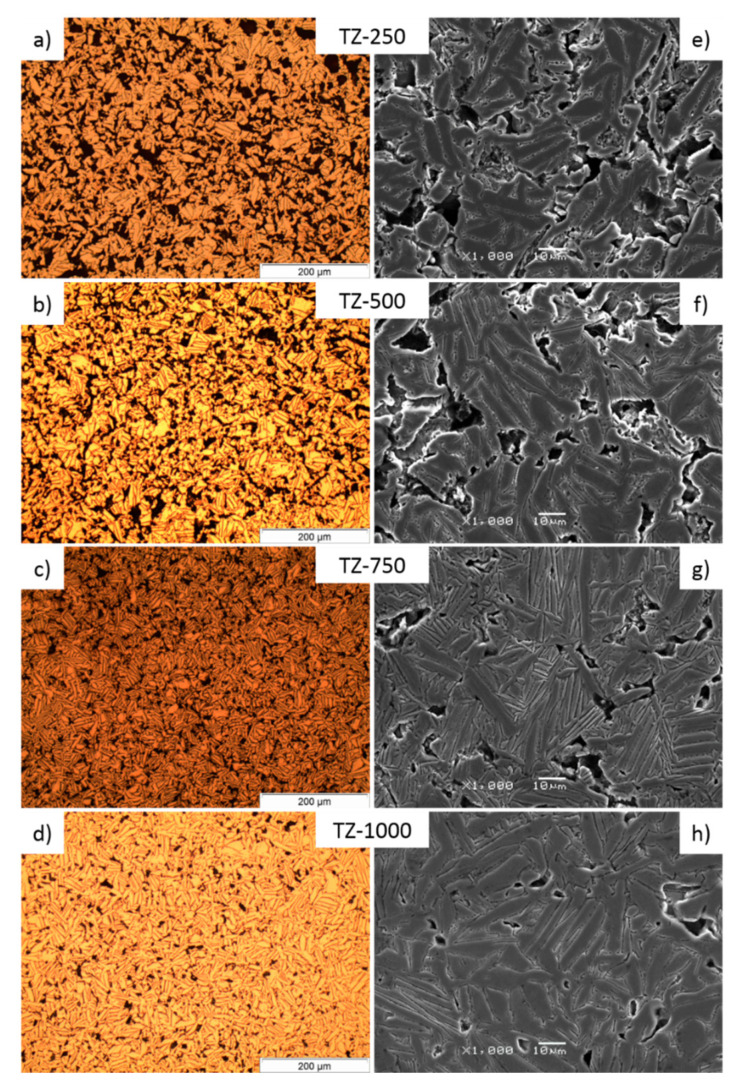
Optical micrographs (**a**–**d**) and SEM micrographs (**e**–**h**) of Ti–35Zr (wt.%) for different pressure during cold isostatic pressuring of 250 MPa (**a**,**e**), 500 MPa (**b**,**f**), 750 MPa (**c**,**g**) and 1000 MPa (**d**,**h**).

The optical and SEM micrographs of the studied samples are presented in Figure 3. For all samples, it was also observed that the particles were connected permanently. The ‘connecting necks’ were created through the diffusion processes during the sintering. The interdiffusion depends a little bit on composition, time and temperature of sintering and also the surface energy. The surface energy per unit volume depends on the inverse of the particle diameter. Thus, smaller particles with high specific surface area have higher energy, so that they could be sintered faster in spite of lower sintering temperature [44,45,46]. Additionally, the microphotographs clearly show progressive changes of the sample morphology according to pressure parameters during the isostatic pressing. All of the Ti–35Zr (wt.%) alloys showed a structure consisting of lamellae and needles, which is typical of α phase. This microstructure is related to the inherent anisotropy of the hexagonal crystal structure of α phase, which is shown by Hsu et al. [40,47]. In the micrographs of the TZ-750 sample, we observe finer lamellae of the grain in comparison to the other samples.

**Figure 4:**
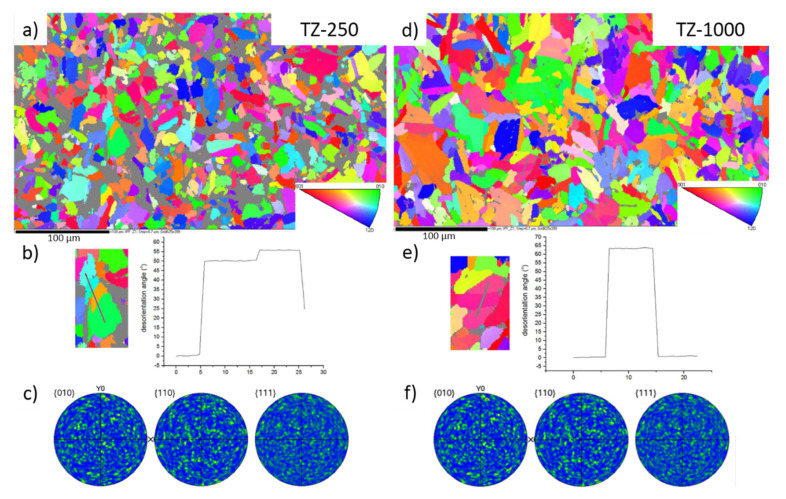
High-resolution electron backscatter diffraction pattern for the samples TZ-250 (**a**) and TZ-1000 (**d**) with representation of disorientation angle between grains for sample TZ-250 (**b**) and TZ-1000 (**e**). Pole figures for the sample TZ-250 (**c**) and TZ-1000 (**f**).

Electron backscatter diffraction measurements confirmed the presence of α phase in all studied samples of the manufactured material. Figure 4a,b,d,e shows the crystallographic orientation maps of the grains for the samples after pressing under the pressure of 250 MPa and 1000 MPa respectively. Grey, unindexed regions correspond to the pores occurring in the studied material. The grain boundary was defined by a misorientation angle higher than 3°. On the EBSD maps, not all grain boundaries observed in the images taken using SEM were marked. After detailed analysis of the EBSD measurements, the low-angle boundaries characterized by the disorientation angle below 3° occurring among the subgrains can also be visible. Such an observation shows that the structure of the material is a hierarchical one, where the bigger grains are composed of a series of smaller, needle-like subgrains. Based on literature reports, the introduction of a hierarchical microstructure on the surface of a titanium implant and supporting it with bioactive elements is a very effective way to improve osseointegration between bone and implant [48,49,50]. Twin boundaries were also observed (Figure 4b,e). Figure 4c,f presents the pole figures calculated from the orientation maps obtained from the EBSD measurements for sample TZ-250 and TZ-1000. An almost statistical distribution of the orientations without the preferred orientations can be visible. The above-mentioned research and the crystallographic orientation maps prove that the obtained material does not exhibit preferred orientation even though it was prepared using cold isostatic pressing.

**Figure 5:**
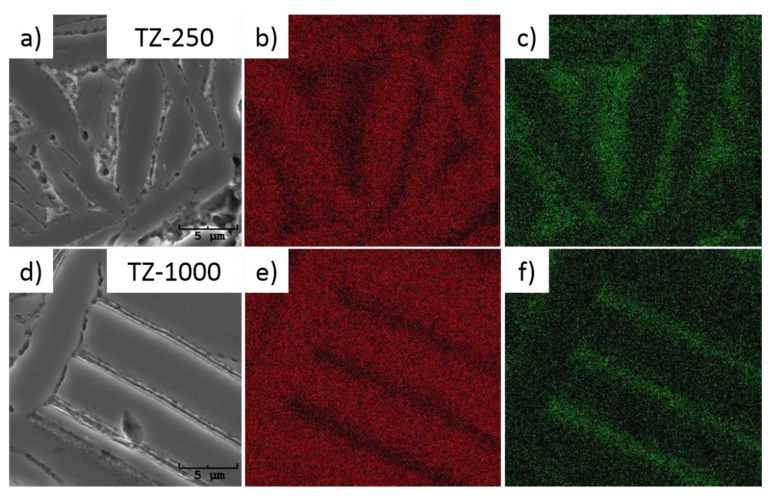
Distribution maps of the elements for the sample under isostatic pressure of 250 MPa (SEM micrograph (**a**), Ti (**b**) and Zr (**c**)) and 1000 MPa (SEM micrograph (**d**), Ti (**e**) and Zr (**f**)).

Chemical elements distribution maps (Figure 5) performed using the EDS detector showed homogeneous distribution of the elements within the grains. However, on the grain boundaries a small decrease of titanium and a small increase of the zirconium can be observed. Such observations can indicate that the surface of the grain is slightly enriched with the zirconium. Also, small holes were observed at the grain boundaries, the location and nature of the cavities suggest that those were formed during the etching process for metallurgical structure observation, and would not influence the corrosion stability [40]. However, there is the possibility that the loss of titanium was also connected with the etching process, because the solution used for etching could be more favorable for boundaries of grains and titanium [51,52,53].

**Figure 6:**
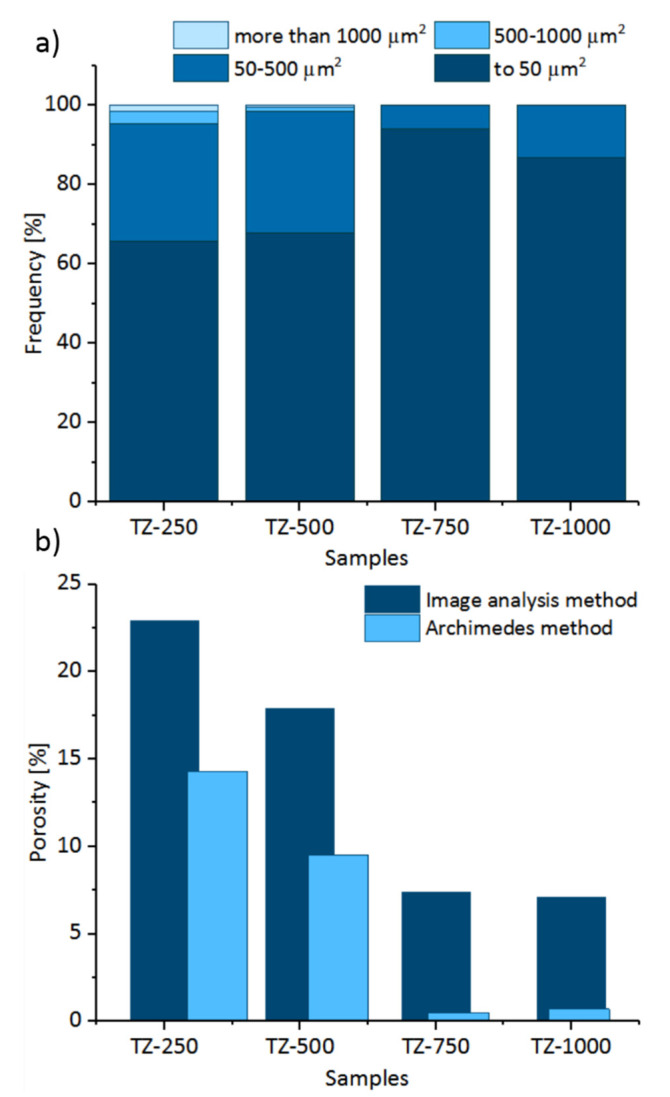
Graphs describing parameters of porosity: percentage of pores in the surface area section within the pre-defined ranges for all samples (**a**) and percentage of pores (**b**) in the sample after pressing with different pressures.

The percentage of the pores (volume fraction) in the observation area was estimated based on that pores surface fraction on the material image taken from an optic microscope. It was found that the degree of porosity is influenced by the pressure of isostatic pressing. For the samples after isostatic pressing under 250, 500, 750 and 1000 MPa, the percentage fraction of the pores was on average 22.9%, 17.9%, 7.4% and 7.1%, respectively. In the literature, the expectation that all the pores are closed when the porosity level is below 6 vol% [54] was checked by the Archimedes method. The Archimedes method demonstrated porosity values that were approximately 6.4–8.6% lower than those obtained by the image analysis (Figure 6b). This is probably due to the partial participation of the closed pores. Production of the porous materials by powder metallurgy makes porosity control possible by applying various pressure forces during isostatic pressing. More significant changes in the average pore cross-sectional area (a_p_) can be observed. An increase in the pressure force during isostatic pressing causes a decrease in the a_p_ parameter. For the samples after different isostatic pressing under 250, 500, 750 and 1000 MPa of pressure, the cross-sectional area of the pores was on average 291.2, 153.7, 21.5 and 34.9 µm^2^, respectively. The average cross-sectional area of the pore for the material after applying the pressure of 250 MPa is over five times higher than that of the case of the pores of the sample treated with the pressure of 750 MPa. Regarding the changes of the maximum cross-sectional area of the pores, for the 250 MPa sample, this value was 4717.91 µm^2^, while for the sample made with the highest pressure maximum cross-sectional area was 372.17 µm^2^. As investigated, it was shown that the maximum pore diameter has a dominant effect on the mechanical properties, given that they have a similar level of density in the compacts [55,56].

A more detailed analysis of the number and size of the pores in the samples is also presented in Figure 6a. The number of pores with a cross-sectional area higher than 500 µm^2^ was the largest for the pressure force of 250 MPa and was about 5% of all pores. The amount of large surface pores gradually decreases when the pressure force of isostatic pressing increases. Such a large cross-sectional area results from the existence of the interconnected pores systems. In the case of materials to be used for implants, interconnection of the pores is of great importance due to the ability of the cells and body fluids to penetrate into the implant.

The analysis of Feret’s diameter (Table 2 and Figure 6a) revealed that for the sample produced under 250 MPa pressure, the pores with diameter larger than 50 µm represent 5% of all pores in this sample. Also, for the sample produced with 500 MPa isostatic pressing, participation of these pores was estimated to be over 3%. Such pore structure can favor osseointegration. For the osseointegration process, the most desirable are those pores with size ranging from 50 µm to several hundred µm [57,58]. The largest proportion of the pores with the cross-sectional area in range 50–500 µm^2^ (Figure 6b) was in the sample manufactured with the application of the pressure of 500 MPa. At the same time, microscopic images show that the systems of interconnected pores still exists. The largest amount of pores with the a_p_ below 50 µm^2^ can be observed for the samples under the pressure of 750 MPa. It is possible that the greater the pressure force, the smaller the amount of pores in this range. It is a result of gradual disappearance of the smallest pores due to the application of a very large pressure force. It can be assumed that we are left with the artefacts of the pores which are smaller than those that can be detected with the use of optical methods. The dimensionless coefficient of circularity (Table 2) increases almost linearly when the applied pressure increases. In this case, the diffusion processes taking place during sintering might be of great significance. The total percentage of pores decreases with increasing pressure while manufacturing material, however other parameters of porosity did not behave linearly depending on pressure. Generally, the pore size in the sintered Ti compacts should decrease with decreasing level of porosity is attributable to the surface energy per unit volume. The porosity level could be controlled by the use of different powder particle size. The shape and size of particles results in green packing density. Furthermore, the space between the coarse powder particles may be filled by the fine powder during preparation of the sample under 750 MPa, which resulted in a different microstructure [45,55,59,60]. As complementary research, BET testes to assess surface area were performed. For the samples TZ-250, TZ-500, TZ-750 and TZ-1000, the surface area was on average 0.71, 0.79, 0.28 and 0.47 m^2^/g respectively. Generally, the obtained values correspond to participation of pores, however the TZ-750 sample revealed the lowest surface of absorption. The prepared research suggests layer-by-layer absorption, which suggested that there could be a layer of oxides on the surface as an effect of sample preparation.

The quantitative analysis of the microstructure, including the determination of the cross-sectional area of the grains and the subgrains as well as the coefficient of grain variability, were performed based on optic microscope images. For the samples TZ-250, TZ-500, TZ-750 and TZ-1000, the average section areas of grains were 35.06 µm^2^, 33.15 µm^2^, 21.17 µm^2^ and 34.85 µm^2^ respectively (Table 3). In terms of the stereological parameter, the sample produced under the pressure of 750 MPa had a different microstructure. The coefficient of the grain variability was also determined. For all the samples this was over 1 (from 1.13 to 1.40). This means that in terms of the size of the cross-sectional area, the resulting structure is strongly diversified. The number of elements that were tested per unit area of the image were also determined and for samples TZ-250, TZ-500, TZ-750 and TZ-1000 were 14,279, 15,967, 25,526 and 3675 (1/mm^2^), respectively. This value correlates with the other analyzed parameters, especially in the case of material made under 750 MPa pressure. The surface adsorption theory can be used to explain the differences of the grain growth, which can be connected with the size of particles or pressure used. The theory of adsorption says that the plane that has higher surface tension grows fastest, while its adsorption amount of surface-active material is maximized. As a result, the surface energy decreases and therefore the growth of this plane reduces [44].

**Figure 7:**
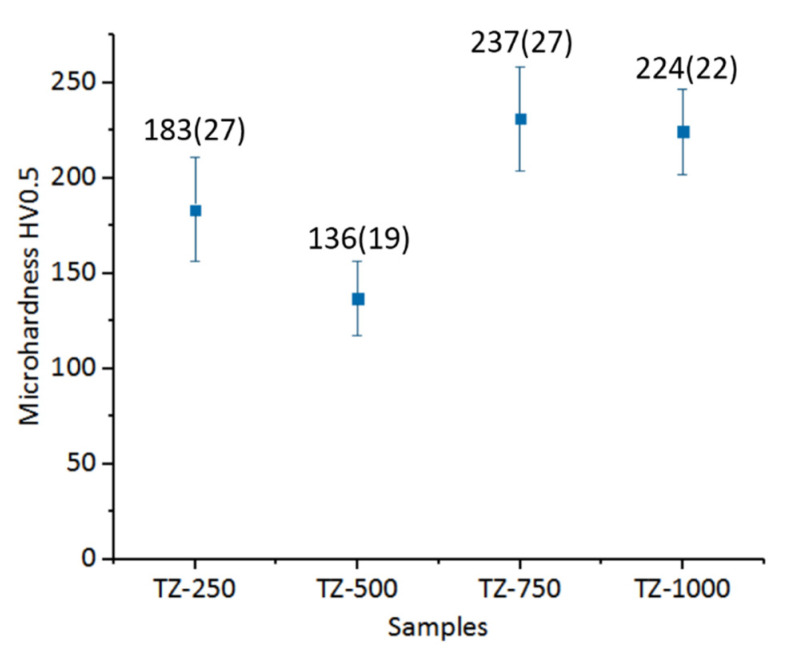
Microhardness values of the obtained materials.

In general, the aim of scientific research is to produce material for which the mechanical properties of the implant are as close as possible to human bone. Studies of mechanical properties carried out on human bone prove that the mechanical properties of bones depend on the type of bone, as well as the type of structure analyzed (compact or spongy). The mean hardness of bone ranged from 33 HV (the head) to 44 HV (the diaphysis). The hardest part of the radius was the shaft, with a value of 42 ± 6 HV. The proximal metaphysis had a hardness value of 34 ± 6 HV, and the distal metaphysis hardness value was 35 ± 5 HV [61,62,63]. Generally, the hardness of material is related to its atomic mobility capacity, distortion of crystalline lattice and atomic displacement. Addition of Zr content caused a crystalline lattice and atomic displacement, which correlate with higher microhardness of alloy [29,30,64]. The measured microhardness (Figure 7) does not exhibit a linear correlation with the applied pressure force. The sample pressed at 500 MPa revealed the lowest value of microhardness, which was 137 HV0.5. Also, for the sample pressed at 1000 MPa, a slight decrease of microhardness in comparison to the sample produced under the pressure of 750 MPa was observed. Compared to Ti–10Zr (wt.%) (266 HV), the results obtained for Ti–40Zr (wt.%) (350 HV) produced by commercial arc-melting vacuum–pressure were lower, which could be connected with the introduction of porosity to samples. What is more, the hardness value of sample prepared under 500 MPa pressure was lower than c.p. Ti (186 HV) [19]. The non-linear behavior of hardness could be connected with grain size, structure of pores or oxygen participation. It is widely known that the mechanical properties of Ti alloys depend essentially on the microstructure. It should be noted that the effect of smaller grain size (i.e., Hall–Patch effect) could reflected in the hardness data since [44], which correlates with the results of stereological analysis of grain size. The literature focuses on the Young’s Module as the most significant parameter in the context of using material for long-term bone implants. Generally, Young’s modulus is characterized by material hardness (understood as resistance to elastic deformation). However, we do not refer to this dependence because there have been measurements of microhardness which do not fully reflect the properties of the whole element, including pores.

## 4. Conclusions

The porous Ti–35Zr (wt.%) alloy has been successfully produced from the mixture of the elemental metal powders. Based on the investigation into the microstructure and mechanical properties, the following main conclusions can be drawn:

Based on the results of the XRD and EBSD methods, all the Ti–35Zr samples showed the α phase. Moreover, the change of pressure after the PM production of the material did not significantly change the unit cell of the α phase.

The material that was analyzed does not reveal the preferred orientation and texture. The low angle boundaries can be seen, which indicate that the structure of the studied material possesses hierarchical character (the bigger grains are composed of a series of smaller subgrains).

The shape and size of the pores, as well as the microhardness value, do not display a linear dependence in relation to the pressure used during the material manufacturing process.

It was found that the most optimal materials for a hypothetical application as implants were those obtained by isostatic pressing of 500 MPa due to the largest number of pores in the cross-sectional area ranging between 50–500 µm^2^ and the presence of the connections between the pores, and also the higher participation of pores with diameter larger than 50 µm.

The pressure of 750 MPa during the manufacturing process of the Ti–35Zr (wt.%) alloys seems to be the threshold value, introducing different morphology of the material as well as the size and shape of the pores. According to conducted structural and microstructural analysis, the samples will be subjected to further research to determine the possibility of using this material as an implant. In the first stage, mechanical properties such as hardness and Young’s modulus of the whole element will be evaluated. The tests of the influence of the PEO process, thermal and alkaline treatments on electrochemical maintenance of samples in simulated physiological solutions (e.g., Tyrode’s solution, Ringer’s solution, artificial saliva) will be carried out. The following fundamental electrochemical techniques will be applied: registration of the open circuit potential (OCP), linear voltammetry (LV), cyclic voltammetry (CV) and electrochemical impedance spectroscopy (EIS). The last stage of research on the material will be biological research. Life testing will be performed using human fibroblasts. For staining, cell cultures will be incubated using fluorescence.

## Figures and Tables

**Table 1:** Lattice parameters of α phase of samples after different isostatic pressure and sintering at 1000 °C for 24 h.

Phase	Lattice Parameters	ICDD *	Samples			
			TZ-250	TZ-500	TZ-750	TZ-1000
α	a_0_ (nm)	0.2951 **	0.3013(2)	0.3011(2)	0.3006(2)	0.3005(3)
	c_0_ (nm)	0.4683 **	0.4783(5)	0.4780(5)	0.4773(5)	0.4772(5)

* International Centre for Diffraction Data^®^; ** ICDD PDF 00-044-1294.

**Table 2:** Results of the Feret’s diameter and circularity of the pores for the samples manufactured under different pressure forces.

Sample	Feret’s Diameter (μm)				Circularity
	Minimum Value	Maximum Value	Average Value	Standard Deviation	Average Value
TZ-250	2.29	218.99	14.84	18.66	0.58
TZ-500	2.17	169.00	12.69	13.10	0.61
TZ-750	2.17	43.44	6.53	4.41	0.70
TZ-1000	2.17	42.86	7.44	5.64	0.70

**Table 3:** Results of the section area of the grains for the samples manufactured under different pressure forces.

Parameter	Sample	Minimum Value	Maximum Value	Average Value	Standard Deviation
a_g_ (µm^2^)	TZ-250	2.03	492.58	35.06	40.99
	TZ-500	2.01	496.13	33.15	44.50
	TZ-750	2.01	474.56	21.17	30.77
	TZ-1000	2.03	492.58	34.85	41.04
